# Modelling mood updating: a proof of principle study

**DOI:** 10.1192/bjp.2022.175

**Published:** 2023-03

**Authors:** James E. Clark, Stuart Watson

**Affiliations:** Translational and Clinical Research Institute, Newcastle University, UK; Translational and Clinical Research Institute, Newcastle University, UK; and Cumbria, Northumberland, Tyne and Wear NHS Foundation Trust, UK

**Keywords:** Bipolar affective disorders, depressive disorders, anxiety disorders, aetiology, cognitive neuroscience

## Abstract

**Background:**

Recent developments in computational psychiatry have led to the hypothesis that mood represents an expectation (prior belief) on the likely interoceptive consequences of action (i.e. emotion). This stems from ideas about how the brain navigates its external world by minimising an upper bound on surprisal (free energy) of sensory information and echoes developments in other perceptual domains.

**Aims:**

In this paper we aim to present a simple partial observable Markov decision process that models mood updating in response to stressful or non-stressful environmental fluctuations while seeking to minimise surprisal in relation to prior beliefs about the likely interoceptive signals experienced with specific actions (attenuating or amplifying stress and pleasure signals).

**Method:**

We examine how, by altering these prior beliefs we can model mood updating in depression, mania and anxiety.

**Results:**

We discuss how these models provide a computational account of mood and its related psychopathology and relate it to previous research in reward processing.

**Conclusions:**

Models such as this can provide hypotheses for experimental work and also open up the potential modelling of predicted disease trajectories in individual patients.

Computational approaches to psychiatric illness attempt to develop mathematical models describing cognitive processes and their mapping to underlying neuronal processes.^1–4^ They have resulted in descriptions of psychosis,^5^ autism^6^ and more recently mood disorders^7^ in terms of false inferences in the brain (see below) and the resulting theories have accumulated significant experimental backing.

In this paper we aim to show that mood states (both healthy and pathological) can be described in terms of basic computational principles. We start with an overview of the principles underlying computational psychiatry and how they might be applied to mood disorders and present a model of mood updating drawing on these principles. The ensuing results are discussed in the context of individual and group phenotyping in mood disorders.

## The brain must infer the likely causes of sensations

The idea that the brain does not have direct access to its external world stems from ancient ideas in philosophy of mind.^8^ This separation between internal and external world is essential if self-organising systems are to maintain a stable internal milieu and avoid the ‘decay to equilibrium’.^9^ This implies the existence of a Markov blanket for such systems^10,11^ – a statistical boundary that induces a conditional independence between internal and external states within a system and separates these systems from a chaotic environment. One consequence of this, however, is that the brain cannot directly access the external world (described as hidden states in the literature on self-organisation) that it must still perceive and navigate – instead it must make inferences based on the information it does have access to: basic sensory data (sensory states) and previous experience of similar encounters (i.e. a specific model of the world). For example, visual and auditory information may not accurately discriminate birds from planes from superheroes; but past experience dictates the object in my line of vision is probably the former. Via a separate line of reasoning the same idea was pioneered by Helmholtz as unconscious (inductive) inference whereby sensations alone are insufficient to explain perception. What is required is an abductive framework in which sensations are interpreted in the context of past experience to explain their likely causes – a process broadly analogous to syllogistic reasoning.^12^

Inferences of this sort are Bayesian and therefore rely on prior beliefs (built from previous experience) about causes of received information (i.e. hidden states). Priors are characterised by their mean (expectation) and precision (inverse variance) and describe *a priori* which hidden states the brain expects to encounter and how certain it is to encounter them. The role of precision in inference is critical. If a prior is very certain then sensations must be highly incongruent to result in a meaningfully different posterior, whereas a very uncertain (vague) prior is highly susceptible to even slight deviations from expectations. Certainty or uncertainty is a function of consistency in previous experiences – so the brain is confident about the likely causes of sensations if the same sensations have previously been related to the same causes. This has important implications for psychopathology as discussed below.

## Prediction errors, entropy and free-energy minimisation

If the brain makes Bayesian inferences about hidden states then we can express the difference between priors and actual events in terms of prediction errors (i.e. what did I think was going to happen versus what actually happened). We can further cast prediction errors in terms of the surprisal associated with events under a given model (past experience). Surprisal affords the brain an opportunity to alter expectations (i.e. to learn) so that expectations are more closely aligned to the most recent events, although the extent to which this occurs is determined by the precision of expectations. This is usually beneficial and allows the brain to categorise events that are broadly similar but that differ slightly in the sensations they cause (as no two events will be completely identical). Too broad a precision would result in overattribution of meaning to events that should be trivial and too narrow would result in novel events being misattributed as familiar (more on this later).

The long-term average of surprisal is entropy, a term that quantifies the brain's uncertainty about hidden states over time. In order to avoid categorisation mistakes as described above, and to successfully navigate hidden states, entropy must be minimised within the constraints of prior experience (in order to limit the dispersion or dissipation of an organism's physiological and belief states^13^). Perceptual inference therefore becomes a problem of minimising surprisal (or optimising evidence for the brain's generative model) associated with hidden states. Unfortunately direct calculation of surprisal is intractable^14^ (it would involve integration over too large a probability space) and instead the brain minimises (or at least appears to minimise) a more tractable upper bound on surprisal, which is termed free energy.^11,15,16^ When fleshed out mathematically we reach an intuitive conclusion that free energy can be minimised (i.e. perceptual inference is made) by either changing expectations so they are more in line with actual events, or by acting to attenuate prediction errors and in doing so maintaining expectations.^17^ Action aimed at fulfilling expectation corresponds to the process of active inference.^18^

## When inference goes wrong

Thus far we have described how the brain minimises free energy in order to infer the hidden states that cause observable sensory input. In the face of prediction errors it does this either by changing expectations or attenuating error signals. However, we noted earlier that the precision of prior beliefs plays a crucial role in this process. If the precision of prior beliefs is too high then novel events (which should change expectations) are ignored and if it is too low then events that should be familiar (and therefore should not alter expectations) can substantially change priors. This explanation for perception and action has led quite naturally to descriptions of hallucinations as perceptual inference based on overly precise priors.^19–21^ This theory supposes that prior beliefs in perceptual stimuli are so strong that resulting inferences are resistant to the empirical absence of such stimuli. In contrast, delusions are the result of imprecise prior beliefs such that sensory attenuation fails and sensations inappropriately alter priors^22^ – phenomenologically this results in the attribution of meaning to events that should be trivial.

## Mood and mood disorders

Thus far discussion has focused on exteroceptive perception, although there is a growing body of evidence that interoceptive inference (perceiving the body's own physiology) plays a crucial role in emotion.^23–26^ According to this view the emotional content of hidden states must be inferred through the interoceptive signals (such as heart rate, adrenaline, cortisol) they cause (note similarities to existing psychological theories of emotion^27^). Active inference can be used to attenuate signals that are discordant with priors and amplify signals that agree with priors. By extending the schema described thus far to hierarchical Bayesian inference we have proposed that mood acts as a hyper-prior (a belief about a belief) over the precision of lower-level perceptual priors.^7^ A more straightforward way of framing this is that mood determines the strength of beliefs about the likely consequences of action. Emotion therefore corresponds to short-term fluctuations in the actual outcomes of our action, whereas mood represents long-term expectations about the emotional states we are likely to encounter. Healthy mood states involve priors that are not overly precise and so allow for changes in mood over time according to actual emotional content of events encountered. For example, in a negative mood state an agent will expect to encounter negative (i.e unsurprising or expected) events but is not resistant to signals that violate these predictions. If they are then to experience positive events their mood will correspondingly become more positive.

Similarly to their role in perceptual abnormalities, priors that are precise or imprecise induce pathological mood states. As such, we have proposed that depression is the result of a precise hyper-prior in negative outcomes of action with ensuing attenuation of contradictory signals. This results in events that should be positive (and would not be so unsurprising in healthy states) being experienced as relatively negative (because they are now unsurprising). Mania represents the opposite state so that events are experienced as positive regardless of their objective emotional content. In contrast, anxiety states represent an imprecise hyper-prior over negative outcomes. Such states prevent action designed to resolve uncertainty (i.e. attenuation of interoceptive signals). As such, we propose that mood states can be described according to their coordinates in a two-dimensional schema characterised by the expectation and precision of hyper-priors on the interoceptive consequences of action.

When we talk about pleasurable and stressful outcomes, we do not imply that an observation is affectively valenced. We simply mean that certain (positive) outcomes are, *a priori*, preferred over other (negative) outcomes. These prior preferences play the role of reward, when it comes to selecting various actions (see below). In other words, a stress signal is simply an observation that an individual does not anticipate encountering, whereas a pleasure signal is a familiar outcome that is, *a priori*, unsurprising (i.e. rewarding).

In this paper we describe a model of mood in which beliefs about the stressful content of hidden states are updated using the formalism described thus far.

## Method

Our model is a partially observable Markov decision process (POMDP) consisting of a state space in which there are:

- two possible states (‘stressful’ and ‘not stressful’); and
- two possible observations (‘stress signals’ and ‘pleasure signals’).

The system can choose from one of five possible actions in each state (‘attenuate pleasure signals’, ‘amplify pleasure signals’, ‘attenuate stress signals’, ‘amplify stress signals’, ‘wait’) that generate observations with a given probability.

**Fig. 1:**
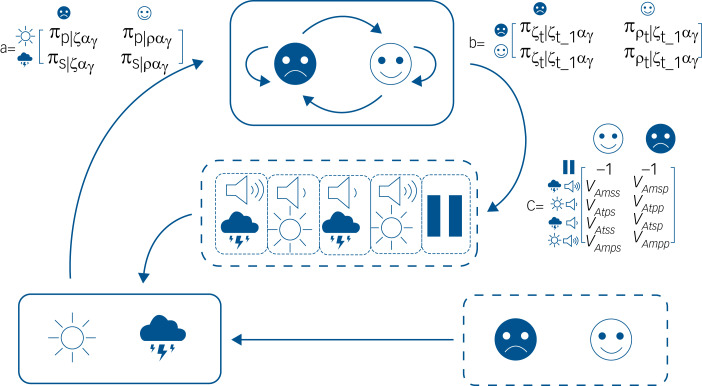
This figure shows the relationship among different states in the model. Observable states are shown in panels with solid lines, whereas hidden states are in panels with broken lines. The environment in this case (shown in the bottom right panel) is either stressful or non-stressful. The system (in this case modelling an agent attempting to infer the emotional content of the environment) will attempt to match their own internal (emotional) states to the environment. The environment generates observations that are either pleasurable (*P* –the sun icon) or stressful (*S* – the storm-cloud). The system must then use these observations to infer the state of the environment and will do so by minimising the difference between its expectations (the ‘mood’ of the system in our model) and the environment. The top panel is, therefore, the internal state (emotion) of the system at any point – again this is either stressful (ζ) or pleasurable (ρ). Matrix (a) is the likelihood matrix and shows the probability that observations are interpreted as stressful or pleasurable under the current internal state and the policy (α_γ_) being followed at the time. The system can transition to a different state or maintain its current state. Whether it does this or not is a function of the policy the system is following at any given time and the state at the previous time point. These probabilities are reflected in the transition probability matrix (b). The policy (or action) of the system is to either amplify or attenuate stress signals according to the optimality function where value is equal to inverse surprisal or model evidence. This is reflected in the reward matrix (c) and highlights the fact that Bellman optimality is a special case of free-energy minimisation. The probability of an observation is conditional on the state of the system and its current policy. The system can either wait, minimise or amplify stress/pleasure signals. Our conjecture is that mood functions as a (hyper)prior distribution over the likely emotional outcomes of any given policy (action). This is best reflected in the probability values in matrix (b), and means that the most valuable policy is the one that minimises the difference between the expected and actual emotional states. This can be achieved either though attenuation or amplification of sensory signals, or by altering the system's own internal states (i.e. changing mood). We propose that a healthy system is relatively receptive to changes in the emotional content of the environment, whereas pathological mood states result in either policy failure or inappropriate policy that results in mood states resistant to environmental signals – as detailed in the main text.

We modelled healthy mood updating, depression, mania and anxiety. The differences between these POMDPs lie in differences in the transition and observation probability matrices (see Fig. 1).

In healthy mood updating the agent is confident that a stressful state will yield stress signals and that non-stressful states will yield pleasure signals. As such, the system will attempt to maintain its belief states (i.e. the probability that an event is stressful or not stressful according to the previous state) but will update them accordingly in the face of conflicting observations. In contrast the depression model was given very strong prior beliefs that it would receive stress signals, regardless of the action it took. The mania model was given very strong prior beliefs that it would receive pleasure signals and the anxiety model was given very weak prior beliefs that it would receive stress signals.

The values used for probabilities are arbitrary but are designed to broadly highlight the underlying principles central to our theories described above. We acknowledge this partitioning of mood states is somewhat simplistic, however, our model should suffice to illustrate the principles discussed above.

It should be noted that our model is a special case of active inference, where policy selection is treated at as a form of planning as inference^28–30^ and policies are selected to minimise expected free energy.^31^ Under some simplifying assumptions (namely there is no intrinsic or epistemic value associated with any policy), expected free energy reduces to expected reward, where reward is the prior (log) likelihood of an outcome. In this special case, one can use the Bellman optimality equation to identify the best action from any given inferred state. In turn, this allows one to use standard POMPD technology to simulate active inference. In this setting the most valuable policy at any point in a POMDP is given by the Bellman optimality equation^32^ (see Supplementary Appendix 1; supplementary material is available at: http://dx.doi.org/10.1192/bjp.2022.175) with reward manually specified according to surprisal.

Directly solving this equation is intractable, and so we use an approximate solution in which continuous belief states are discretised in a grid and a parameterised convex combination sought that necessarily provides an upper boundary on the true optimal value function.^33–35^ This boundary is then minimised to find the most appropriate approximate solution.

All the models were run in the POMDP package^36^ in the R statistical environment^37^ and the output presented as a network in which nodes correspond to belief states and edges the transitions between them if certain signals are received. Within each node the optimal action for the system at that time is also presented. Networks were constructed using the Qgraph package in R.^38^ We also present the frequency density plot of each belief state in the network and the bargraph corresponding to the frequency with which the model is is each belief state. R code used can be found in Supplementary Appendix 2.

## Results

**Fig. 2:**
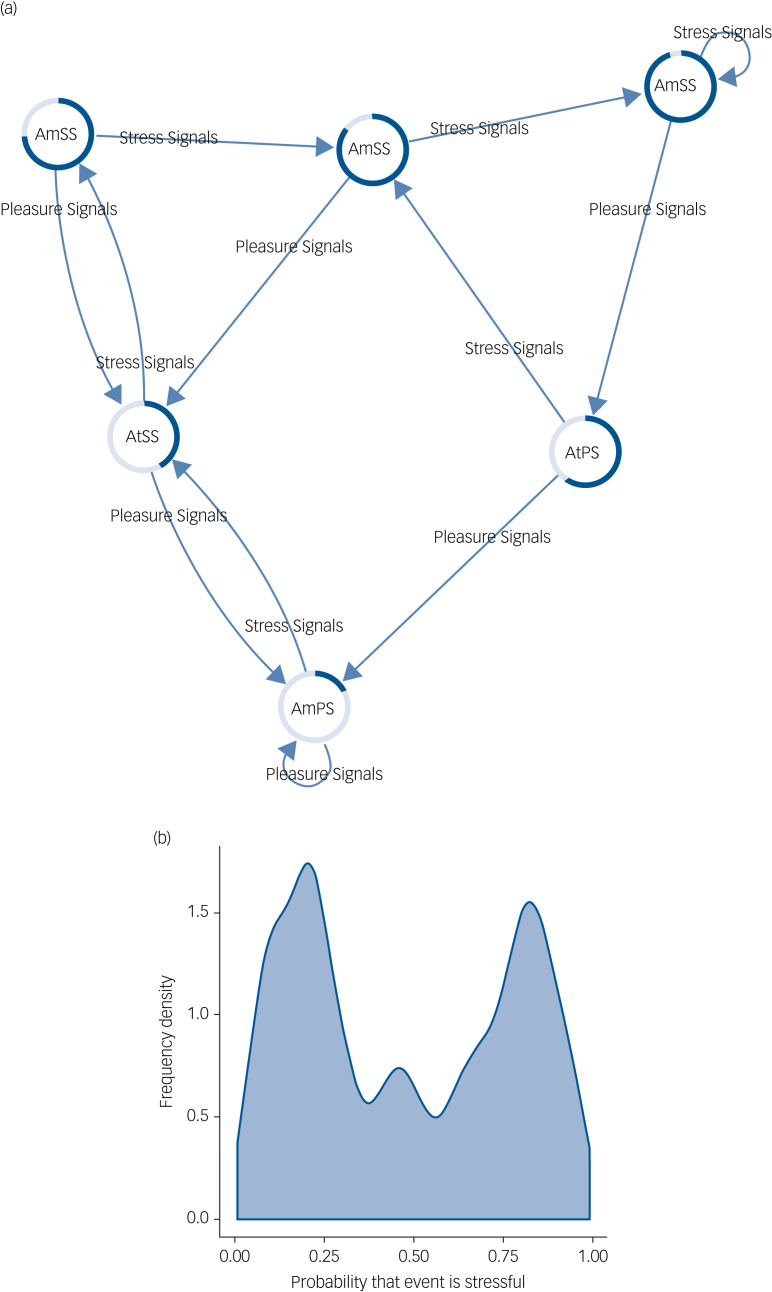
(a) This network shows how belief states about the stress content of the environment are updated in a healthy mood state. Arrows indicate transitions between belief states based on the type of signals the agent observes. The coloured edges of each node represent the probability that the environment is stressful (dark blue portion) or non-stressful (light blue portion). Text within each node represents the optimal action that the agent will take given the current belief state. (b) This figure shows a frequency density plot of the probability that an event is decided to be stressful by the agent in healthy mood updating. Note that there is roughly equal density shared between being certain an event is non-stressful and certain an event is stressful. The key to the healthy mood network, therefore, is an ability to transition between mood states and a resistance to uncertainty about outcomes of action (the region in the middle of the distribution). AmSS, amplify stress signals; AtSS, attenuate stress signals; AmPS, amplify pleasure signals; AtPS, attenuate pleasure signals.

The network of healthy mood updating is presented in Fig. 2(a). There are six nodes representing various degrees of certainty/uncertainty that the event encountered is stressful. Note that when the agent is very certain in a particular outcome it attempts to amplify signals that correspond to its current belief state, although when it is less certain, the optimal policy switches to attenuating belief-inconsistent signals. Importantly, in this model belief states are altered in line with overwhelming evidence with the aim of producing certainty in current mood states. This results in the frequency density graph shown in Fig. 2(b) that reflects an agent capable of visiting mood states between stressful and not stressful, with reasonable confidence.

Interestingly, the agent seems to show pleasure-seeking behaviour, spending most time confident it will encounter a non-stressful environment, although (crucially) this does not preclude an appropriate mood response to received stress signals. Supplementary Table 1 shows the belief distributions for each node in the network.

**Fig. 3:**
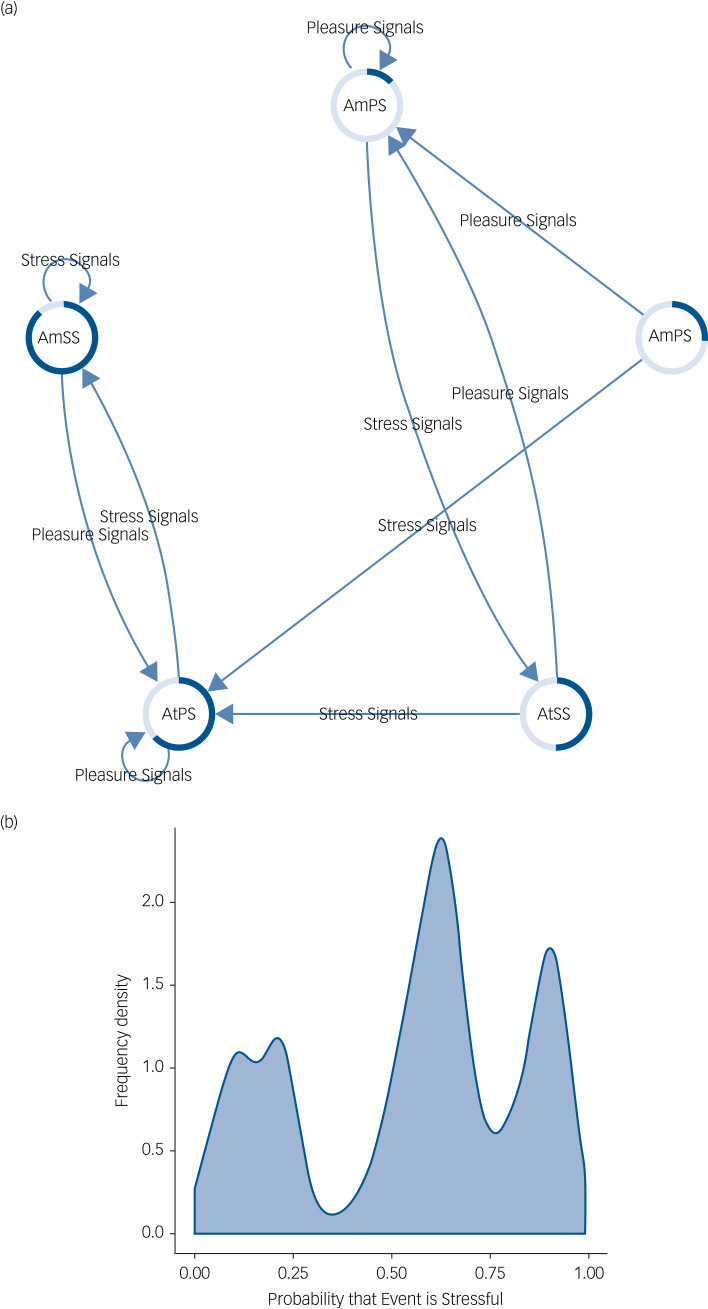
(a) This network shows how belief states about the stress content of the environment are updated in a depressed mood state. Arrows indicate transitions between belief states based on the type of signals the agent observes. The coloured edges of each node represent the probability that the environment is stressful (dark blue portion) or non-stressful (light blue portion). Text within each node represents the optimal action that the agent will take given the current belief state. Note that if enough stress signals are received the agent becomes stuck in a loop in which belief states are constantly expecting a stressful environment and action is aimed at maintaining this belief, despite conflicting signals. (b) This figure shows a frequency density plot of the probability that an event is inferred to be stressful by the agent in depressed mood updating. In this case the distribution is skewed to the right (in contrast to Fig. 2(b)) indicating a much greater frequency of a stressful environment. AmSS, amplify stress signals; AtSS, attenuate stress signals; AmPS, amplify pleasure signals; AtPS, attenuate pleasure signals.

This is in contrast to the modelled depressed system. We see in Fig. 3(a) that this agent still experiences events as not stressful, although if stress signals are received the belief states of the agent have a permanent expectation of stress. This is to such an extent that if pleasure signals are received they are attenuated until stress signals are received, resulting in a highly certain belief that events are stressful. The resultant frequency density graph is skewed towards increased probability of stressful outcomes (Fig. 3(b)) with most time spent in stressful belief states.

**Fig. 4:**
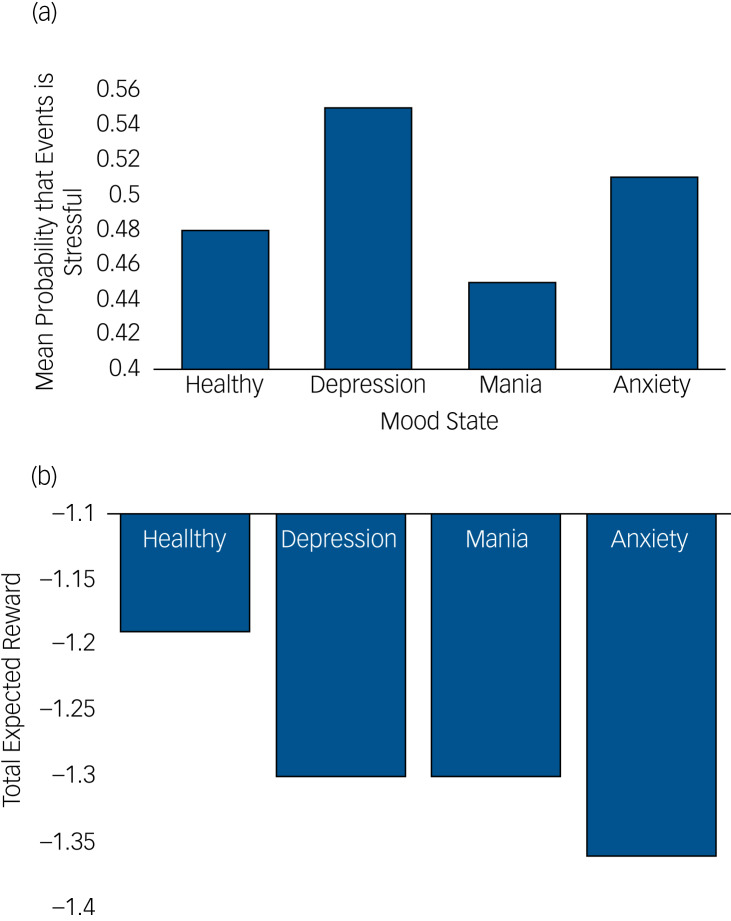
(a) Bar graph showing mean probability in each mood state, across all belief states, that the agent thinks an event is stressful. The healthy mood state shows a more balanced probability, whereas in depression and anxiety the agent is more likely to believe events are stressful. In mania, events are more likely labelled as non-stressful. (b) Bar graph showing total expected reward from most valuable policy in each mood state. Depression and mania are associated with lower rewards, although not as low as the anxiety state. This is because reward in this context is framed in terms of minimising surprisal (maximising model evidence) that relies on certainty in belief states.

The probability that an event will be experienced as stressful in this case is much greater than in the healthy model (Fig. 4(a)). Supplementary Table 2 shows the belief distributions for each node in the network.

**Fig. 5:**
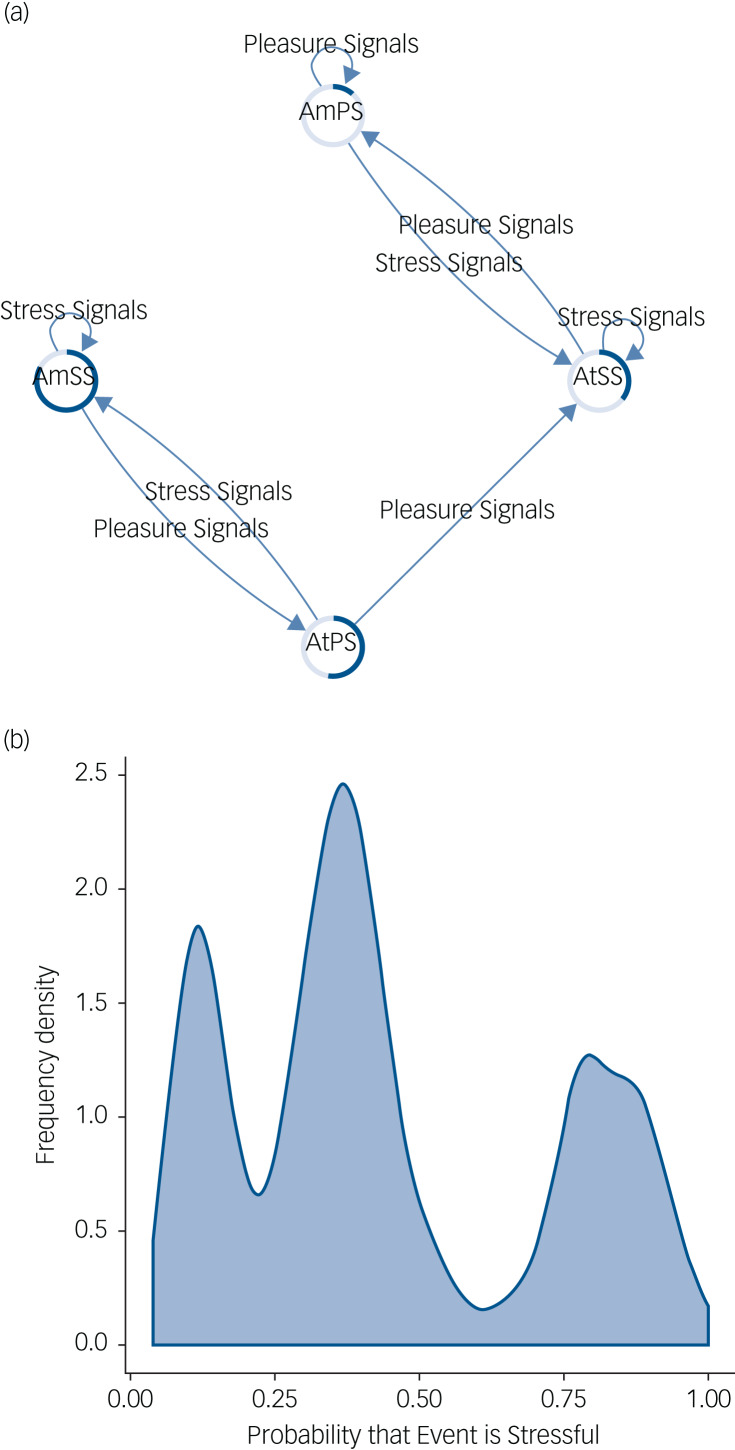
(a) This network shows how belief states about the stress content of the environment are updated in a manic mood state. Arrows indicate transitions between belief states based on the type of signals the agent observes. The coloured edges of each node represent the probability that the environment is stressful (dark blue portion) or non-stressful (light blue portion). Text within each node represents the optimal action that the agent will take given the current belief state. Note that if enough pleasure signals are received the agent becomes stuck in a loop in which belief states are constantly expecting a non-stressful environment and action is aimed at maintaining this belief, despite conflicting signals. (b) This figure shows a frequency density plot of the probability that an event is inferred to be stressful by the agent in manic mood updating. In this case the distribution is skewed to the left (in contrast to Fig. 1(b)) indicating a much greater frequency of a non-stressful environment. AmSS, amplify stress signals; AtSS, attenuate stress signals; AmPS, amplify pleasure signals; AtPS, attenuate pleasure signals.

The model of mania was essentially the opposite of the depression model whereby consistent pleasure signals resulted in a loop in which stress signals are attenuated and a belief state in a non-stressful outcome is maintained (Fig. 5(a)). Ensuing graphs shows increased probability that events will be experienced as not stressful (Figs. 4(a) and 5(b)). Supplementary Table 3 shows the belief distributions for each node in the network.

**Fig. 6:**
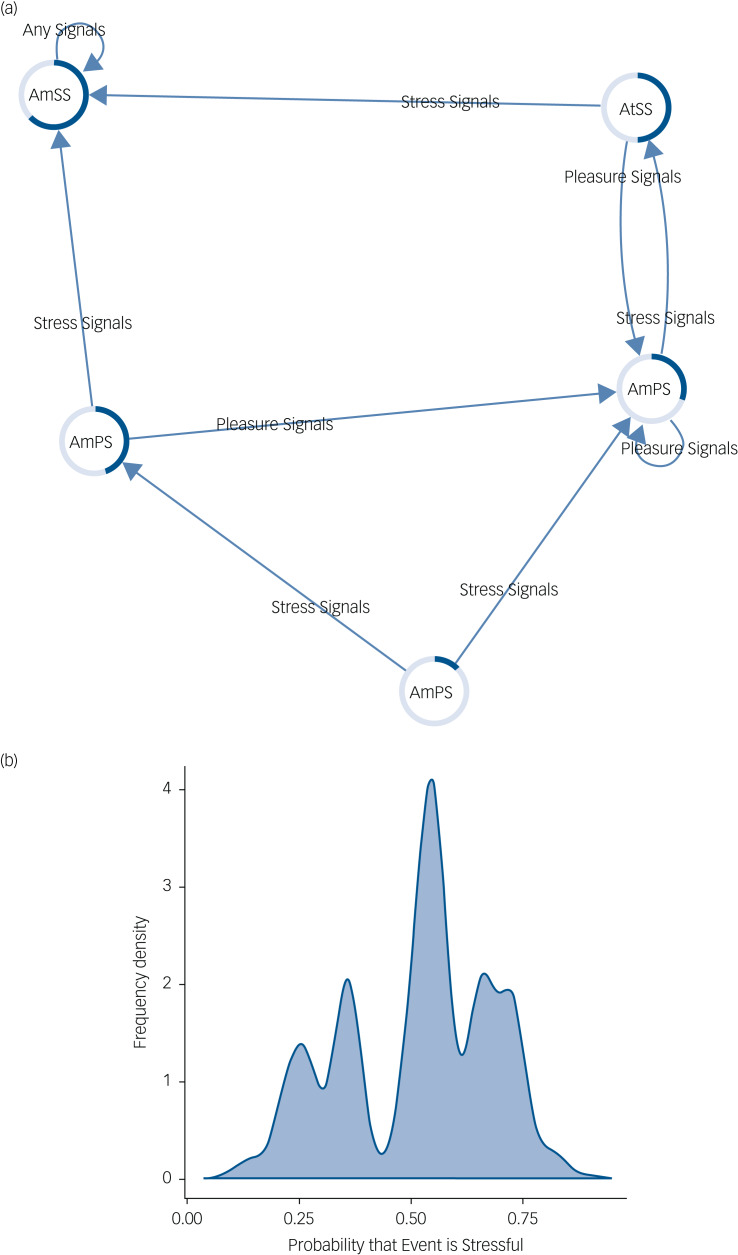
(a) This network shows how belief states about the stress content of the environment are updated in the anxiety mood state. Arrows indicate transitions between belief states based on the type of signals the agent observes. The coloured edges of each node represent the probability that the environment is stressful (dark blue portion) or non-stressful (light blue portion). Text within each node represents the optimal action that the agent will take given the current belief state. In this case nodes are generally much more uncertain. Note that, unlike the other models, the agent attempts to amplify belief-consistent signals under uncertainty. Eventually, if enough stress signals are received, the agent becomes stuck in a node characterised by uncertainty about a stressful environment that is maintained whichever signals are received. (b) This figure shows a frequency density plot of the probability that an event is inferred to be stressful by the agent in anxious mood updating. In this case the distribution is quite normal with a peak at an uncertain belief in a stressful outcome. We propose this inability to resolve uncertainty is central to anxiety states. AmSS, amplify stress signals; AtSS, attenuate stress signals; AmPS, amplify pleasure signals; AtPS, attenuate pleasure signals.

The anxiety network (Fig. 6(a)) was characterised by much more uncertainty. Although there are still belief states in where non-stressful expectations are held, if the agent experiences enough stress signals it becomes stuck in a final node characterised by relative imprecision in expected outcomes. Crucially, action in the active inference framework requires precise beliefs hence the self-reinforcing nature of this loop. This is reflected in a frequency density with a peak at low stressful probability, but with comparatively wider tails (Fig. 6(b)). Interestingly, the probability of an event being interpreted as stressful was lower than that found in the depression network, although still higher than that in the healthy agent (Fig. 4). Supplementary Table 4 shows the belief distributions for each node in the network.

Total expected reward (framed in terms of negative surprisal or model evidence) was highest in the healthy agent, although both depression and mania yielded greater rewards than anxiety (Fig. 4(b)).

## Discussion

### Main findings

In this paper we have presented a POMDP of healthy mood updating, framed in terms of active inference, and subsequently compared it with models of depression, mania and anxiety. The ensuing simulations show that healthy mood involves amplifying signals consistent with highly certain belief states, and attenuating signals inconsistent with uncertain beliefs – although it is crucially able to fluctuate between interpretation of events as stressful and not stressful with some certainty. In contrast the agent with depression, after experiencing significant stress, became resistant to the typically belief-altering effects of pleasure signals. Similarly, mania resulted in belief states resistant to stress signals. Modelled anxiety resulted in generally uncertain belief states, and significant stress resulted in a highly uncertain belief in stressful outcomes that was characterised by failure of action to resolve uncertainty and was therefore maintained despite any type of signal.

### Interpretation of our findings

Recent theoretical work has proposed that we can frame mood, computationally, as prior beliefs about the likely consequences of action (i.e. emotion) with mood disorders represented at the extrema of expectations and certainty about these priors. Our results support this. One particularly interesting feature of the healthy model was the value placed in amplifying belief-consistent signals when belief states were certain, and attenuating belief-inconsistent signals when belief states were uncertain. There are multiple studies of exteroception showing an increase in bottom-up signal amplification when outcomes are predictable^39–41^ whereas sensory attenuation is reserved to resolve uncertainty. The same phenomena are yet to be explored fully in interoception although they have been hypothesised.^23,42^ Intuitively, these findings suggest that behaviour aimed at fulfilling expectations is more likely when such expectations are certain whereas attenuation, in contrast, is used in times of uncertainty in an attempt to maintain current belief states. If both forms of action fail to suppress prediction error then expectations must change.

By altering the agent's predictions about the effects of action on hidden and sensory states we were able to model pathological mood states. In our model of depression, the agent was relatively certain that whatever action it took it would result in a stressful outcome. This meant that once it experienced sufficient stress signals it entered a loop in which pleasure signals could only reduce certainty in a stressful hidden state until they were attenuated and certainty regained. This corresponds with evidence that brain reward-learning signals in patients with depression are blunted, whereas punishment signals are enhanced.^43–45^ Interestingly, the distinction between attenuation and amplification of signals is also important here, with recent work suggesting patients show hyposensitivity to reward, but not hypersensitivity to punishment,^46^ although the latter phenomenon has been observed elsewhere.^47^

Interestingly, in the anxiety model prolonged stress induced a state of uncertain belief in stressful events (in line with our theoretical work) that was maintained regardless of further signals received. In this case the agent attempted to amplify stress signals, rather than attenuate pleasure signals – in contrast to the other models tested. Failure of sensory attenuation is likely to be a key mechanism in the maintenance of a highly uncertain belief state and intuitively represents a failure to rule out possible but unexpected outcomes of action (equivalent to a type 2 error in statistical reasoning). These types of errors in reasoning have been reported in healthy individuals with high trait anxiety during a target-identification task^48,49^ with some evidence that patients with anxiety disorders have exaggerated responses to interoceptive signals.^50,51^ Our results are in line with these findings and support the idea that a key difference between anxiety and depressed states is failure of interoceptive attenuation with resulting prolonged uncertainty about emotional states.

We also found that expected reward (framed in terms of expected model evidence or negative surprisal) was greatest in the healthy model. It was, however, also lower in the anxiety model compared with the depressed or manic model. Recall that the frequency density plots above suggest that healthy mood updating is characterised by certainty in expected outcomes (be they positive or negative) and the agent spends very little of its time in an uncertain belief state. Expected reward in mood disorders will correspondingly be lower in systems that spend more time in uncertain belief states – as is reflected in our results. A key observation here is that mood disorders are the product of Bayes optimal behaviour in terms of maximising expected reward in the context of altered prior beliefs about the outcomes of action. Therapeutic approaches will therefore necessarily result in suboptimal behaviour (relative to current internal models) if priors (initially) remain unaltered. In this sense it is interesting that antidepressants induce rapid changes in emotional processing despite taking longer to alter mood.^52^ This observation may also have particular relevance in treatment resistant disorders that, intuitively, may be characterised by especially certain priors.

Ultimately, the type of model we have presented may prove useful in modelling disease trajectories in groups of patients – or potentially individual patients. This, however, would require a conceptual leap in experimentally quantifying belief states. Heuristic approaches in psychosis have used mismatch negativity blood-oxygen-level dependent (BOLD) responses or time spent observing novel information to model the processing of uncertainty although this does not provide values for exact belief states. One possibility is to establish how such belief states are biologically encoded in the pharmacology and connectivity of neuromodulator systems. Our model predicts that brains must encode (hyper)priors in their functional anatomy. Specifically, expectations will correspond to top–down drives of neuromodulator systems while precision is encoded in the gain control afforded to ascending prediction error signals. This may explain common findings in systems such as the hypothalamus–pituitary–adrenal axis in mood and mood disorders^53–55^ but requires formal testing. This could be done, for example, by comparing serial cortisol measurements in response to expectation violation in patients and controls and examining changes under various pharmacological conditions (for example corticosteroid receptor blockade). Establishing the biological correlates of belief states in healthy and pathological mood would provide a reasonable method to evaluate various (POMDP) models for best fit in specific groups or possibly individuals.

### Implications

In this paper we have discussed recent theoretical developments in computational psychiatry that aim to describe mood and, therefore, mood disorders in terms of prior beliefs about the likely consequences of action. We used a POMDP model of various mood states to show how agents in various mood states might update their beliefs under such a formulation. Our results showed that healthy mood updating was characterised by switching between relative certainty in stressful and non-stressful outcomes with sensory attenuation employed as an optimal policy when belief states were uncertain. In contrast, depression and mania were characterised by inappropriate attenuation to maintain relatively fixed belief states whereas anxiety was characterised by inappropriate uncertainty that precluded previously optimal attenuation strategies.

We have discussed our results in relation to existing findings and elaborated on future developments that this approach might yield. Ultimately, the aim of this type of modelling strategy would be to model group, or even individual, disease trajectories with greater accuracy than existing models.

## Data Availability

Data availability is not applicable to this article as no new data were created or analysed in this study.

## Supplementary material

For supplementary material accompanying this paper visit https://doi.org/10.1192/bjp.2022.175. click here to view supplementary material
