# Supplementary material for: Modelling mood updating: a proof of principle study
Source: Br J Psychiatry. 2023 Mar;222(3):125–34. doi: 10.1192/bjp.2022.175 (PMC9929713; doi:10.1192/bjp.2022.175)
Supplement: Supplementary file 1 [file S0007125022001751sup001.zip › Appendix_1.docx]

Appendix 1

# Partially Observable Markov Decision Processes

A partially observable Markov decision process (POMDP) is a seven tuple:

$$\left\langle S,A,T,R,Z,O,\gamma\right\rangle$$

$S$ is a set of hidden states. In this paper the states are “*stressful*” and “*non-stressful*”.

$A$ is a set of possible actions the agent might take. In this paper these are “*amplify stress signals*”, “*attenuate stress signals*”, “*amplify pleasure signals*”, “*attenuate pleasure signals*” and “*wait*”.

$T$ is a transition matrix of prior probabilities in moving between states under a particular action. This varied by mood state (shown in supplemental material). Specific values were chosen in arbitrary way to highlight the principles of mood updating in each mood state.

$R$ specifies reward, which is a function of action and new state. In our model reward was specified by the surprisal (negative log probability) of each outcome given $T$. The result is that the agent, in each mood state, must infer the best policy to allow it to minimise surprisal of observations under prior beliefs about the consequences of action.

$Z$ is a set of possible observations. In this case: “*stress signals*” or “*pleasure signals*”.

$O$ is a probability matrix specifying prior beliefs about the probability of a particular observation given a specific action and resulting hidden state. Again, this changes with each modelled mood state.

$\gamma$ is a discount factor between 0 and 1 which determines whether the agent will behave in a short-sighted (maximise immediate expected rewards) or long-sighted (maximise long term expected rewards) way. Recent evidence suggests this have an important role in pathological mood states though, to maximise simplicity, it is set at 0.5 for each of the models in this paper.

Belief states $(b)$ are probability distributions which are updated according to the following rule:

$\tau\left( b,a,z \right)\left( s^{'} \right)= \frac{\sum_{s\in S} P\left( z,s^{'}|s,a \right)b(s)}{P(z|b,a)}$

A POMDP aims to model the behaviour of an agent whose goal is to work out an optimal set of actions (known as a policy) to maximise expected rewards given the probability matrices specified in the model. The optimal policy is given by the Bellman value function:

$$V^{*}\left( b \right)= \max_{a\in A} \left[ r\left( b,a \right)+ \gamma\sum_{z\in Z} P\left( z|b,a \right)V^{*}\left( \tau\left( b,a,z \right) \right) \right]$$

Exactly calculating the value function is intractable (as there are too many possible belief states) and so several methods for approximation have been developed. In this paper we use a grid based approximation. A grid (G) is imagined in which belief states are discretised to produce a finite number of belief states and a convex value function calculated based on these new states:

$$\hat{V}\left( b \right)= \sum_{i=1}^{|G|} \lambda(i)\hat{v}(b_{i}^{G})$$

This function is necessarily an upper boundary on the true value function. As such, a belief state (given as a convex combination of grid points) is chosen which minimises the above equation (and therefore provides the best approximation of the true value function).

This approach has interesting parallels with the process of minimising free energy, which necessarily provides an upper boundary on surprisal.
