# Supplementary material for: Modelling mood updating: a proof of principle study
Source: Br J Psychiatry. 2023 Mar;222(3):125–34. doi: 10.1192/bjp.2022.175 (PMC9929713; doi:10.1192/bjp.2022.175)
Supplement: Supplementary file 1 [file S0007125022001751sup001.zip › Supplementary_Material_Tables.docx]

# Supplementary Tables

# Table 1

| Node | Probability of stressful hidden state | Probability of non-stressful hidden state |
| --- | --- | --- |
| One | 0.17 | 0.83 |
| Two | 0.42 | 0.58 |
| Three | 0.60 | 0.40 |
| Four | 0.74 | 0.26 |
| Five | 0.86 | 0.14 |
| Six | 0.95 | 0.05 |

**Table 1 Legend**: Belief state for each node in the healthy mood network. Values refer to the probability that the hidden state is stressful or non-stressful.

# Table 2

| Node | Probability of stressful hidden state | Probability of non-stressful hidden state |
| --- | --- | --- |
| One | 0.13 | 0.87 |
| Two | 0.27 | 0.73 |
| Three | 0.50 | 0.50 |
| Four | 0.63 | 0.37 |
| Five | 0.89 | 0.11 |

**Table 2 Legend**: Belief state for each node in the depressed mood network. Values refer to the probability that the hidden state is stressful or non-stressful.

# Table 3

| Node | Probability of stressful hidden state | Probability of non-stressful hidden state |
| --- | --- | --- |
| One | 0.12 | 0.88 |
| Two | 0.36 | 0.64 |
| Three | 0.53 | 0.47 |
| Four | 0.83 | 0.17 |

**Table 3 Legend**: Belief state for each node in the manic mood network. Values refer to the probability that the hidden state is stressful or non-stressful.

# Table 4

| Node | Probability of stressful hidden state | Probability of non-stressful hidden state |
| --- | --- | --- |
| One | 0.13 | 0.87 |
| Two | 0.31 | 0.69 |
| Three | 0.45 | 0.55 |
| Four | 0.50 | 0.50 |
| Five | 0.63 | 0.37 |

**Table 4 Legend**: Belief state for each node in the anxiety mood network. Values refer to the probability that the hidden state is stressful or non-stressful.
